# Risk of de novo post-transplant type 2 diabetes in patients undergoing liver transplant for non-alcoholic steatohepatitis

**DOI:** 10.1186/s12876-015-0407-y

**Published:** 2015-12-15

**Authors:** Maria Stepanova, Linda Henry, Rishi Garg, Shirley Kalwaney, Sammy Saab, Zobair Younossi

**Affiliations:** 1 Center for Liver Diseases, Department of Medicine, Inova Fairfax Hospital, Falls Church, VA USA; 2 Betty and Guy Beatty Center for Integrated Research, Inova Health System, Claude Moore Health Education and Research Building, 3rd floor, 3300 Gallows Road, Falls Church, VA 22042 USA; 3 Department of Medicine, David Geffen School of Medicine at the University of California at Los Angeles, Los Angeles, CA USA

**Keywords:** Liver transplantation, Type 2 diabetes, NASH

## Abstract

**Background:**

Non-alcoholic steatohepatitis (NASH) is often seen together with components of metabolic syndrome. The aim of this study was to assess the risk of de novo post-transplant type 2 diabetes (DM) in liver transplant recipients with NASH.

**Methods:**

All adult patients from the Scientific Registry of Transplant Recipients (2003–2012) transplanted for NASH or cryptogenic cirrhosis (the NASH cohort) without pre-transplant DM were included in this retrospective cross-sectional study.

**Results:**

Total 2,916 NASH subjects and 14,268 controls with non-HCV related cirrhosis or hepatocellular carcinoma were included. Patients with NASH were, on average, 6 years older, more likely female and overweight/obese. By 5 years post-transplant, 39.8 % NASH vs. 27.0 % controls developed at least one onset of de novo DM; this was observed starting 6 months post-transplant: 22.9 % vs. 16.7 % (relative risk 1.38). Later in follow-up, the relative risk of de novo DM was also higher in NASH: 1.46 by 3 years, 1.47 by 5 years (all *p* < 0.0001). After exclusion of DM that resolved after the first year, long-term DM remained higher in the NASH cohort: 7.6 % vs. 4.3 %, *p* < 0.0001. In multivariate analysis, after adjustment for confounders including the use of immunosuppressants, having NASH was independently associated with development of de novo post-transplant DM: adjusted hazard ratio (95 % CI) = 1.29 (1.18–1.42), *p* < 0.0001.

**Conclusions:**

Liver transplant recipients with NASH have a higher risk of de novo post-transplant DM. This suggests the presence of an underlying metabolic disorder beyond fatty liver that may be causative for both NASH and type 2 diabetes.

## Background

The prevalence of non-alcoholic fatty liver disease (NAFLD) is increasing along with the worldwide epidemics of obesity and related conditions such as type 2 diabetes (DM) and metabolic syndrome [1–5]. Although NAFLD describes a broad spectrum ranging from simple hepatic steatosis to non-alcoholic steatohepatitis (NASH), it is only those with NASH that are most likely to progress to cirrhosis, hepatocellular carcinoma and, ultimately, liver failure [1–3]. The overall rate of progression to end-stage liver disease is estimated to be as low as 2-3 % of non-cirrhotic NAFLD patients and up to 13 % in cirrhotic NASH [4, 5]. However, given the size of affected population - the prevalence of NAFLD in the U.S. is estimated to be in the range of 30–50 % [6, 7], − the incidence of end-stage liver disease due to NASH can still be substantial and, more importantly, increasing. Indeed, U.S. transplant centers have recently reported a rapidly growing number of patients who are now receiving liver transplant because of the etiology of their liver disease being NASH [8, 9].

It is estimated that up to 90 % of NAFLD patients may exhibit at least one component of metabolic syndrome such as obesity, insulin resistance or type 2 diabetes, hyperlipidemia, and cardiovascular disease [4, 10]. In fact, the overlap of NAFLD and metabolic syndrome is so extensive that NAFLD is sometimes referred to as hepatic manifestation of metabolic syndrome [11]. Although metabolic syndrome components such as type 2 diabetes are known to be risk factors for progression of NAFLD to more advanced forms [12, 13], the causal relationship between the two is likely complex and also involves other factors such as increased visceral adipose tissue mass and excessive inflammatory signaling. The precise pathophysiologic mechanisms that link NAFLD and type 2 diabetes are not completely clear, but are currently believed to include altered proinflammatory adipo-cytokine production, increased free fatty acid release and the resulting oxidative stress [4, 7]. Furthermore, a number of genetic factors that could potentially drive both insulin resistance and NAFLD progression have already been reported [7, 14–17].

After successful liver transplantation, the underlying metabolic disorder that initially caused progression to NASH and to end-stage liver disease may not resolve and, thus, may still promote both recurrent NASH and metabolic syndrome components in transplanted patients [6, 18, 19]. Indeed, high rate of NASH recurrence in such patients has already been reported: as many as 40 % to 60 % of NASH patients developed graft steatosis as early as 1 year post-transplant [6, 18, 19]. On the other hand, the rate of post-transplant DM in solid organ transplant recipients is high regardless of etiology of their end-stage organ failure, solely due to immunosuppression with diabetogenic agents such as corticosteroids and tacrolimus [20, 21]. Given that, it is unclear whether the risk of post-transplant DM in liver transplant recipients with NASH is additionally affected. However, we suggest that the pathophysiologic mechanism responsible for the initial development and further recurrence of NASH, genetic or environmental, may also contribute to an increased risk of developing DM after receiving liver transplant.

The aim of this study was to compare the rates of developing de novo post-transplant DM in patients transplanted for NASH and in those transplanted for other causes of end-stage liver disease.

## Methods

In this study, we used data from the Scientific Registry of Transplant Recipients (SRTR) released on June 1, 2013. The SRTR data system includes data on all donor, wait-listed candidates, and transplant recipients in the US, submitted by the members of the Organ Procurement and Transplantation Network (OPTN), and has been described elsewhere. The Health Resources and Services Administration (HRSA), U.S. Department of Health and Human Services provides oversight to the activities of the OPTN and SRTR contractors.

Included were liver transplant recipients of 18+ years of age at the moment of transplant who were discharged alive after receiving a heterotopic or an orthotopic liver transplant from a deceased or a living donor. Patients with pre-transplant history of type 2 diabetes were excluded; thus, only de novo episodes of DM were studied. We also excluded patients of less than 18 years of age at the time of transplant, patients receiving liver re-transplants, and patients who died before discharge.

Liver transplant recipients with NASH listed as an indication for liver transplant with or without liver malignancy were included in the target cohort. Since in the United States cryptogenic cirrhosis is largely presumed to be burned out NASH [22–29], we also considered all subjects with an indication for liver transplantation being cryptogenic cirrhosis to have had NASH. Additionally, since hepatitis C virus (HCV) is known to have a diabetogenic effect of its own [30, 31], we also excluded all subjects with HCV-related indication for transplantation or positive HCV serology. Finally, the control non-NASH cohort included subjects with all other causes of chronic liver disease, including alcoholic cirrhosis, hepatitis B, biliary cirrhosis, and metabolic disorders.

### Post-transplant type 2 diabetes

The primary outcome used in this study was time to development of de novo post-transplant DM in liver transplant recipients who did not have DM before transplantation and who were discharged alive after receiving the transplant. Post-transplant follow-up records submitted to the SRTR by UNOS were used to assess post-transplant diabetes status. Since regular reporting of diabetes status in post-transplant follow-up did not start until 2003, only patients transplanted between 2003 and 2012 were included in this study. Patients were followed-up 6 months after transplantation and then annually until death, graft failure, or loss to follow-up. Reporting of diabetes status in liver transplant recipients by UNOS was largely discontinued after 5 years post-transplant, so post-transplant DM was assessed up to 5 years only.

In patients with at least two follow-up records, resolution of post-transplant DM was presumed in those who had a recorded onset of DM followed by a recorded absence of DM in a later follow-up. On the other hand, long-term DM was defined as DM reported in at least two consecutive yearly follow-ups. Time from the transplant to the first onset of DM and from the first onset to its resolution was calculated in years; the follow-up yearly codes were used.

### Study definitions

As reported in the SRTR data collection, transplant recipients' age, gender, ethnicity, citizenship, education, employment status, and the primary payer (public insurance included Medicare, Medicaid, VA, or another government-sponsored plan) were recorded. Functional status at the moment of transplant was reported on a 10–100 scale; the highest value of 100 indicates the best possible health with no evidence of any disease, and the lowest value of 10 indicates moribund status with fatal processes progressing rapidly. Being obese at the time of transplant was defined as having BMI of at least 30.0; overweight was defined as BMI between 25.0 and 30.0. Pre-transplant history of hypertension, peripheral vascular disease, coronary artery disease (CAD), stroke, cancer (solid organ or lymphoproliferative), pulmonary embolism, chronic obstructive pulmonary disease was also recorded.

In addition to having NASH, the transplant diagnosis of primary liver malignancy was defined as having hepatocellular carcinoma, fibrolamellar hepatocellular carcinoma, cholangiocarcinoma, hepatoblastoma, hemangioendothelioma, hemangiosarcoma, angiosarcoma, and other types of primary liver malignancy.

The MELD score for the study cohort was calculated using labs collected before transplantation or, if unavailable, the last record of the wait-listed candidate was used. Furthermore, post-transplant length of inpatient stay was calculated from the date of transplant to the date of discharge; the total length of stay was calculated from the date of admission to the date of discharge.

The use of immunosuppressive medications including mycophenolate (mycophenolate mofetil or mycophenolate sodium, branded or generic), tacrolimus (branded original, modified release, or generic), and steroids (prednisone, methylprednisolone, branded or generic) was recorded at the moment of transplant and at every follow-up.

### Statistical analysis

Clinico-demographic parameters of NASH patients were compared to those of the controls using chi-square test for homogeneity and Wilcoxon non-parametric test. Predictors of time to development of de novo post-transplant DM were evaluated using a multivariate Cox proportional hazard model; the time to development of DM was calculated from the transplant to the first recorded DM onset, and censoring time was the last recorded absence of DM. In order to account for the effect of immunosuppressive drugs on development of post-transplant DM, the use of immunosuppressants was recorded at or before the first onset of DM; if the first use of a particular immunosuppressant happened later than the first onset of DM, such use was not recorded. Two-sided p-values of 0.05 or less were considered statistically significant after Bonferroni multiple test correction.

All statistical analyses were run in SAS 9.3 (SAS Institute, Cary, NC).

### Ethics statement

The study was granted approval by Inova Institutional Review Board.

## Results

In 2003–2012, 5079 adult patients with NASH with at least one follow-up record were transplanted for the first time and discharged from 114 different transplant centers across the U.S. (both transplant centers and patients were de-identified in the SRTR database). Of those, 2916 were reported to have no type 2 diabetes before transplantation, and were used in this study. Similarly, of non-NASH non-HCV controls, 17,447 adult patients receiving their first liver transplant were initially selected, and then 14,268 without pre-transplant history of type 2 DM were included in this study.

The number of NASH transplants without pre-transplant DM per year ranged from 169 in 2003 to 374 in 2011. In the NASH cohort, 1,723 (59.1 %) were in fact transplanted for cryptogenic cirrhosis. Of non-NASH controls, 29.6 % had alcoholic liver disease, 6.6 % had chronic hepatitis B, 6.5 % had metabolic disease such as α1-antitrypsin deficiency, Wilson’s disease or hemochromatosis, 9.7 % had biliary cirrhosis (primary or secondary), 12.7 % had primary sclerosing cholangitis, 6.1 % had autoimmune hepatitis, and 15.4 % had primary liver malignancy of unspecified etiology. Of all patients included in this study, 62.3 % were followed-up for at least 3 years, and 39.6 % for 5 years.

The demographics of NASH transplant recipients and controls is summarized in Table 1. Patients with NASH were, on average, 6.0 years older, significantly more likely Caucasian (81.6 % vs. 72.8 %), less likely male (56.1 % vs. 60.1 %), African-American (4.0 % vs. 9.0 %) or Asian (1.7 % vs. 6.9 %), more frequently covered by a publicly sponsored health insurance plan (Medicare, Medicaid, etc.: 39.2 % vs. 33.4 %), and less likely employed at the time of transplant (13.3 % vs. 17.7 %) (all *p* < 0.0001). The functional status that reflected the severity of disability at the moment of transplant was similar between NASH patients and controls (*p* > 0.05) (Table 1).

**Table 1:** Adult liver transplant recipients with NASH without pre-transplant DM and controls

Parameter	NASH w/o DM	Controls w/o DM	*p*
N	2916	14,268	
Transplant years (N):			
2003–2004	396 (13.6 %)	2371 (16.6 %)	
2005–2006	536 (18.4 %)	2716 (19.0 %)	
2007–2008	610 (20.9 %)	3058 (21.4 %)	
2009–2010	690 (23.7 %)	3113 (21.8 %)	
2011–2012	684 (23.5 %)	3010 (21.1 %)	0.05
Age, years	56.8 ± 10.4	50.8 ± 12.7	<0.0001
Race/ethnicity:			
Caucasian	2378 (81.6 %)	10,384 (72.8 %)	<0.0001
African-American	116 (4.0 %)	1289 (9.0 %)	<0.0001
Asian	51 (1.7 %)	986 (6.9 %)	<0.0001
Hispanic	352 (12.1 %)	1466 (10.3 %)	0.0041
Other race	19 (0.7 %)	143 (1.0 %)	0.07
Male gender	1637 (56.1 %)	8581 (60.1 %)	0.0001
U.S. citizen	2840 (97.4 %)	13,660 (95.7 %)	<0.0001
College degree	657 (26.2 %)	3509 (29.1 %)	0.0031
Primary pay:			
private insurance	1751 (60.3 %)	9308 (65.7 %)	<0.0001
public insurance	1139 (39.2 %)	4737 (33.4 %)	<0.0001
self-pay	12 (0.4 %)	126 (0.9 %)	0.0091
Employed	341 (13.3 %)	2119 (17.7 %)	<0.0001
Functional status (10–100 scale)	58.4 ± 23.9	58.4 ± 26.0	0.26

Clinical presentation of NASH transplant recipients as opposed to controls is summarized in Table 2. Patients with NASH were expectedly more overweight or obese (77.5 % vs. 57.6 %). Despite not having pre-transplant DM, they also had higher prevalence of metabolic syndrome components and related conditions including drug-treated hypertension (23.4 % vs. 17.2 %) and coronary artery disease (3.1 % vs. 1.5 %) (all *p* < 0.0001). The last pre-transplant MELD score was, on average, 0.7 points higher in patients with NASH compared to controls (Table 2).

**Table 2:** Pre-transplant clinical history and transplantation of liver transplant recipients with NASH without pre-transplant DM

	NASH w/o DM	Controls w/o DM	*p*
BMI	30.1 ± 6.3	26.7 ± 5.6	<0.0001
Overweight (BMI 25–30)	871 (30.6 %)	4,608 (33.0 %)	0.0128
Obese (BMI > =30)	1,333 (46.9 %)	3,427 (24.6 %)	<0.0001
Chronic obstructive pulmonary disease	29 (1.2 %)	164 (1.4 %)	0.47
Medically-treated hypertension	574 (23.4 %)	2,062 (17.2 %)	<0.0001
Coronary artery disease	76 (3.1 %)	184 (1.5 %)	<0.0001
Stroke	21 (0.8 %)	77 (0.6 %)	0.24
Peripheral vascular disease	24 (1.0 %)	108 (0.9 %)	0.73
Pulmonary embolism	8 (0.3 %)	45 (0.4 %)	0.72
Malignancy (solid organ or lymphoproliferative)	210 (7.2 %)	1,544 (10.9 %)	<0.0001
Other solid organ transplants	7 (0.2 %)	106 (0.7 %)	0.0022
Primary liver malignancy	130 (4.5 %)	2,202 (15.4 %)	<0.0001
TIPSS	300 (10.4 %)	1,073 (7.6 %)	<0.0001
Transplantation			
Last MELD score	22.8 ± 8.5	22.2 ± 9.9	<0.0001
Heterotopic transplant	3 (0.1 %)	15 (0.1 %)	0.97
Transplant from a living donor	97 (3.3 %)	729 (5.1 %)	<0.0001
Donor’s age	43.2 ± 17.4	41.4 ± 17.6	<0.0001
Donor’s history of DM	315 (11.2 %)	1,357 (10.1 %)	0.06
Procurement from a non-heart beating donor	137 (4.9 %)	572 (4.2 %)	0.13
Number of HLA mismatches with a donor	4.52 ± 1.15	4.55 ± 1.14	0.39
Immunosuppressants used at transplant:			
Mycophenolates	2,275 (78.2 %)	10,943 (76.9 %)	0.14
Tacrolimus	2,695 (92.6 %)	13,235 (93.0 %)	0.43
Steroids	2,763 (94.9 %)	13,519 (95.0 %)	0.88
Rejection episode before discharge	166 (6.2 %)	999 (7.8 %)	0.0031
Post-transplant inpatient stay, days	16.0 ± 18.7	15.6 ± 19.9	0.0498
Total inpatient stay, days	20.7 ± 36.2	20.1 ± 29.2	0.16
Non-compliant in follow-up (ever)	104 (4.0 %)	764 (6.0 %)	0.0001

Patients with NASH were slightly less frequently transplanted from living donors (3.3 % vs. 5.1 %). The use of immunosuppressive drugs was similar between NASH and controls (*p* > 0.05) (Table 2).

### De novo post-transplant type 2 diabetes

During post-transplant follow-up (average duration 3.0 ± 1.7 years), 39.8 % of NASH patients with at least one follow-up record and without history of pre-transplant DM vs. 27.0 % of controls had at least one documented episode of de novo DM (*p* < 0.0001). Of those, 65.9 % and 67.4 %, respectively, had their first onset of DM recorded at the 6 months follow-up, and 79.0 % vs. 80.2 % had it by their 1 year follow-up.

**Fig. 1:**
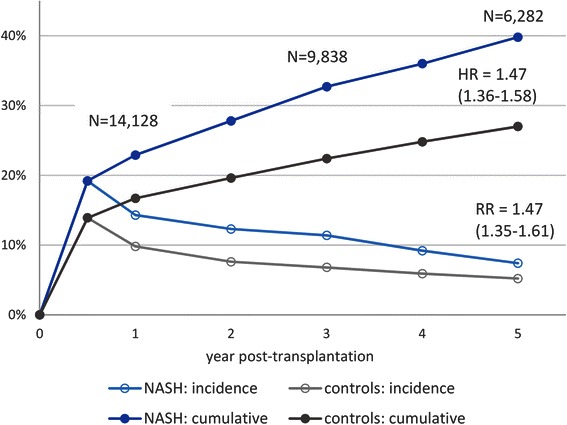
Development of de novo post-transplant type 2 diabetes in liver transplant recipients with NASH vs. controls

Higher rate of de novo post-transplant DM in NASH patients as compared to controls was observed starting as early as 6 months post-transplant: 19.2 % vs. 13.9 % (*p* < 0.0001). Later in follow-up, both cumulative and incidental risks of developing post-transplant DM were again consistently higher in NASH patients (Table 3, Fig. 1). Indeed, 1 year after liver transplantation, the relative risk (RR) of having had at least one episode of de novo DM was RR (95%CI) = 1.37 (1.27–1.49); the same risks by 3 and 5 years post-transplant were similarly significant: 1.46 (1.35–1.57) and 1.47 (1.35–1.61), respectively (all *p* < 0.0001). Furthermore, throughout follow-up, NASH patients were found to be, on average, 1.47 times more likely to develop de novo DM post-transplant in any given time period (hazard ratio (HR) for time to development of DM: 1.47 (1.36–1.58), *p* < 0.0001 (Fig. 1).

**Table 3:** De novo type 2 diabetes at follow-up of liver transplant recipients with NASH

	NASH w/o DM	Controls w/o DM	Relative risk (95 % CI)	*p*
Incidence of de novo DM at:				
6 months follow-up	499 (19.2 %)	1,737 (13.9 %)	1.38 (1.26–1.51)	<0.0001
1 year follow-up	344 (14.3 %)	1144 (9.8 %)	1.46 (1.31–1.64)	<0.0001
3 years follow-up	186 (11.4 %)	560 (6.8 %)	1.67 (1.43–1.96)	<0.0001
5 years follow-up	73 (7.4 %)	275 (5.2 %)	1.43 (1.12–1.84)	0.0046
Had at least one onset of de novo post-transplant DM by:				
1 year follow-up	624 (22.9 %)	2,196 (16.7 %)	1.37 (1.27–1.49)	<0.0001
3 years follow-up	578 (32.7 %)	2,004 (22.4 %)	1.46 (1.35–1.57)	<0.0001
5 years follow-up	425 (39.8 %)	1,548 (27.0 %)	1.47 (1.35–1.61)	<0.0001
Recorded resolution of post-transplant DM after the first onset	621 (73.8 %)	2,205 (74.7 %)	0.99 (0.94–1.03)	0.59
Time from onset to resolution, years	1.13 ± 1.06	1.07 ± 0.99		0.35
Long-term de novo post-transplant DM	174 (7.6 %)	481 (4.3 %)	1.76 (1.49–2.08)	<0.0001

In multivariate analysis with a Cox proportional hazard regression model (Table 4), after accounting for potential risk factors such as higher rate of non-DM metabolic syndrome components at pre-transplant, being transplanted for NASH was still found to be independently associated with an increased risk of development of de novo post-transplant DM: aHR (95 % CI) = 1.29 (1.18–1.42), *p* < 0.0001. Other predictors of de novo DM in post-liver transplant patients included older age (aHR = 1.017 (1.013–1.020) per year), African-American race (aHR = 1.32 (1.16–1.51), reference category: Caucasian), male gender (1.11 (1.03–1.19)), being overweight (1.13 (1.04–1.24)) or obese (1.32 (1.21–1.45)) at transplant, and receiving steroids for immunosuppression at transplant or later in follow-up (but no later than the first onset of DM was recorded) (1.87 (1.49–2.34)) (all *p* < 0.01). On the other hand, the use of other immunosuppressive medications such as tacrolimus and mycophenolates were not associated with post-transplant DM (both *p* > 0.05) (Table 4).

**Table 4:** Predictors of development of de novo post-transplant type 2 diabetes (adjusted hazard ratio with 95 % confidence interval is calculated using Cox proportional hazard model). Total N used in the model = 13,000: n_c_ = 9999 censored, n_e_ = 3,001 events (de novo DM onset)

Predictor	aHR (95 % CI)	*p*
NASH	1.29 (1.18–1.41)	<.0001
Calendar year	0.92 (0.91–0.94)	<.0001
Age at transplant, per year	1.02 (1.01–1.02)	<.0001
African-American	1.32 (1.16–1.51)	<.0001
Hispanic	1.11 (0.99–1.24)	0.08
Asian	0.99 (0.84–1.17)	0.92
Male gender	1.11 (1.03–1.19)	0.0080
Overweight	1.13 (1.04–1.24)	0.0061
Obese	1.32 (1.21–1.45)	<.0001
Liver malignancy	0.96 (0.86–1.08)	0.49
Pre-transplant CAD	1.19 (0.92–1.53)	0.19
Pre-transplant hypertension	1.05 (0.96–1.15)	0.27
Donor’s age, per year	1.002 (1.000–1.004)	0.0414
Procurement from a non-heart-beating donor	1.24 (1.04–1.46)	0.0140
Donor’s history of DM	1.09 (0.96–1.22)	0.18
Use of tacrolimus *)	0.98 (0.83–1.15)	0.80
Use of mycophenolates *)	0.92 (0.84–1.02)	0.11
Use of steroids *)	1.87 (1.49–2.34)	<.0001

*) Ever used before the first onset of post-transplant DM

**Fig. 2:**
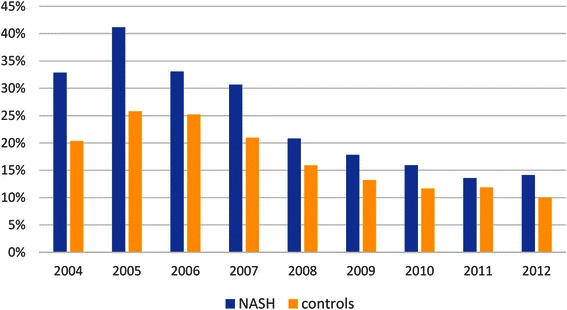
One year post-transplant prevalence of de novo type 2 diabetes in NASH patients and in controls by year. *p* < 0.05 for all years except for 2011

Calendar year was associated with lower risk of developing de novo post-transplant DM: aHR = 0.923 (0.910–0.935) per year. Indeed, of the study cohort transplanted in 2004, 16.3 % developed DM at 6 months post-transplant and 22.3 % by 1 year follow-up, while the same rates for patients transplanted in 2011 were 10.1 % and 12.2 %, respectively (both *p* < 0.0001) (Fig. 2).

### Resolution of de novo post-transplant type 2 diabetes

Of patients who developed de novo post-transplant DM and had at least two follow-up records, 74.5 % had resolution of their DM later in follow-up. The rate of DM resolution was similar between NASH patients and controls (*p* > 0.05), and so was the time to resolution: 1.13 ± 1.06 years vs. 1.07 ± 0.99 years (*p* = 0.35). On the other hand, the rate of long-term post-transplant DM (DM that persisted for longer than 1 year and thus was less likely to be immunosuppression-related) was approximately 75 % higher in NASH patients: 7.6 % vs. 4.3 %, RR = 1.76 (1.49–2.08) (Table 3).

## Discussion

Non-alcoholic fatty liver disease is considered to be hepatic manifestation of metabolic syndrome, a condition that is growing rapidly worldwide synchronously with the epidemic of obesity. As a result, its progressive form of NASH has already become the second most common indication for liver transplantation in the United States after hepatitis C infection and alcoholic liver disease [8, 9, 32].

Tightly connected with other metabolic syndrome components, NAFLD and NASH pose substantial societal burden due to both associated risk of progression to advanced stages and also their role in exacerbation of other metabolic conditions such as insulin resistance and type 2 diabetes. A number of potential pathophysiologic mechanisms that link NAFLD to IR/DM bi-directionally based on the combination of insulin desensitization with systemic and hepatic inflammation have been suggested [4]. It is, however, unknown, whether the increased risk of DM persists in NASH patients after receiving a liver transplant. On the other hand, current understanding of pathophysiology of both NAFLD and insulin resistance suggests that, although both conditions likely serve as direct risk factors for each other, it is a systemic disorder that actually drives both and, thus, is likely to remain in place even after successful liver transplantation.

In this study of a national registry of solid organ transplant recipients, we analyzed the rate of de novo post-transplant DM in patients transplanted for NASH who did not have a recorded history of type 2 diabetes before transplantation, and compared that to controls with non-HCV-related chronic liver disease, since other indications for liver transplantation are not expected to be driven by any systemic IR-related abnormalities. Due to limitations of the SRTR data collection used in this study, only patients with up to 5 years of follow-up transplanted after 2003 were included. Pediatric patients were also not included in this study, although the rapidly growing rate of childhood obesity and associated NAFLD suggests that this cohort may become an important contributor to the overall burden of NASH-associated end-stage liver disease in the nearest future [33].

Our results confirm the increased risk of developing post-transplant type 2 diabetes in patients transplanted for NASH. Although the incidence of DM was found to be decreasing over time regardless of liver disease etiology, both incidental and cumulative risks of de novo post-transplant DM remained consistently higher in the NASH cohort throughout the duration of follow-up. In fact, patients transplanted for NASH had, on average, 35–60 % higher risk of developing de novo post-transplant DM at any moment after transplantation. Furthermore, NASH patients had an approximately 65 % higher risk of developing DM that would last for more than 1 year, indicating that even after accounting for intense immunosuppression shortly after transplantation, which is known to be a major contributor to developing post-transplant DM by itself, NASH patients still remain at higher risk of DM.

Although patients transplanted for NASH also had other medical conditions that could potentially be responsible for an increased risk of having DM, such as obesity, even after adjustment for the baseline confounders, the association of pre-transplant NASH and de novo post-transplant DM remained significant. On the other hand, other predictors of de novo post-transplant DM reported in this study are consistent with previous reports and include older age, male gender, African-American race and being overweight or obese before transplantation. Another important predictor of lower risk of post-transplant DM was calendar year, suggesting that post-transplant management of liver transplant recipients is rapidly improving.

Our results have important clinical implications. In particular, in addition to all health risks associated with DM itself, having DM post-transplant was found to be associated with an increased risk of post-transplant mortality after accounting for a number of potential confounders [34, 35]. Furthermore, given the high rate of NASH recurrence in transplanted patients and that NASH may be both driven by and at the same time increase the risk of post-transplant DM, with longer follow-up, a higher risk of graft loss and the need to re-transplant is also possible. Therefore, post-transplant management of NASH patients, including the choice of immunosuppressants, should account for those increased risks in order to reduce the rate of unfavorable outcomes.

To the limitations of this study, we could not fully assess the effect of post-transplant immunosuppressive medications on development and resolution of post-transplant DM because they were reported without dosage or other details of the regimen, thus, limiting our ability to accurately account for their diabetogenic effect. We also had to rely on candidates’ medical records to rule in and rule out the presence of type 2 DM before transplantation, rather than use universal diagnostic criteria, which may be not absolutely accurate due to variability in coding practices across transplant centers.

## Conclusion

In conclusion, in this study of the nationwide registry of patients receiving liver transplantation, we have confirmed that, in addition to previously known risk factors, patients transplanted for NASH are at a greater risk of developing de novo post-transplant DM even in the absence of type 2 diabetes before transplantation. This suggests the presence of an underlying metabolic disorder beyond fatty liver that may be causative for developing of both NASH and DM, and should guide clinicians for long-term management of patients undergoing liver transplantation for NASH.

## Abbreviations

aHR: adjusted hazard ratio
ALD: alcoholic liver disease
CAD: coronary artery disease
CI: confidence interval
HRSA: Health Resources and Services Administration
MMRF: Minneapolis Medical Research Foundation
NAFLD: non-alcoholic fatty liver disease
NASH: non-alcoholic steatohepatitis (NASH)
OPTN: Organ Procurement and Transplantation Network
RR: relative risk
SRTR: Scientific Registry of Transplant Recipients
